# Long-Term pulmonary function outcomes in survivors of congenital diaphragmatic hernia

**DOI:** 10.1007/s00383-026-06316-7

**Published:** 2026-02-13

**Authors:** Carolin Riemer, Philipp Utz, Florian Kipfmueller, Thomas Schaible, Eda Yildirim, Meike Weiss, Greta Thater, Christoph Mohr, Kaja Riebesell, Jana Hoffmann, Michael Boettcher, Michaela Klinke, Julia Elrod

**Affiliations:** 1 Department of Pediatric Surgery, University Medical Center Mannheim, University of Heidelberg, Mannheim, Germany; 2 Department of Pediatrics, University Medical Center Mannheim, Pediatric Pulmonology and Allergy Associates, University of Heidelberg, Hirschberg, Germany; 3 Department of Neonatology, University Medical Center Mannheim, Heidelberg University, Mannheim , Germany; 4 Department of Neurosurgery, Heidelberg University Hospital, University of Heidelberg, Mannheim, Germany; 5 Department of Clinical Radiology and Nuclear Medicine, University Medical Center Mannheim, Heidelberg University, Mannheim , Germany

**Keywords:** CDH, Congenital diaphragmatic hernia, Pulmonary function, Obstructive, Restrictive

## Abstract

**Background:**

Congenital diaphragmatic hernia (CDH) is a rare developmental defect associated with pulmonary hypoplasia and long-term respiratory morbidity. Data on pulmonary function trajectories and structural predictors in CDH survivors remain limited. Objective of this study was to quantify long-term pulmonary dysfunction and its predictors in survivors of congenital diaphragmatic hernia (CDH).

**Methods:**

A prospective single-centre cohort of CDH survivors who underwent pulmonary function testing (PFT) between 2013 and 2023 was analysed. A total of 338 PFTs from 260 patients (57.7% male; birth years 2000–2017) were included; body plethysmography was available in 156 cases. Thoracic morphology was assessed by computed tomography or magnetic resonance imaging performed within six months of PFT. Spirometric and plethysmographic parameters were evaluated using z-scores derived from Global Lung Function Initiative reference equations. Associations with clinical characteristics, neonatal course, surgical technique, and imaging-derived thoracic indices were analysed using non-parametric testing, correlation analysis, and multiple linear regression.

**Results:**

Obstructive ventilatory impairment occurred in 24.6% (mild 20.6%, moderate 44.4%, severe 22.2%); restrictive ventilatory impairment in 22.4% (mild 51.4%, moderate 40.0%, severe 8.6%). Lower zFEV₁ was associated with prenatal diagnosis, right-sided hernia, large defect, liver herniation, extracorporeal membrane oxygenation (ECMO), open repair and patch closure (all *p* ≤ 0.003). Regression (R² = 0.32) showed + 0.51 zFEV₁ per kg birth weight; negative coefficients for large defect (-0.39), right-sided hernia (-0.57), ECMO (-0.64) and open repair (-0.97). Haller index > 2.7, correction index ≥ 0.1 or asymmetry index outside 0.95–1.05 further reduced zFEV₁ and/or zTLC (*p* ≤ 0.03). During 3–5-year follow-up (*n* = 50), lung function stabilised or improved in 82%, but progressive obstruction developed in 18%.

**Conclusion:**

Approximately one quarter of CDH survivors exhibit persistent ventilatory deficits; defect severity, intensive therapy and chest-wall deformity independently predict poorer outcomes. The high incidence of long-term pulmonary impairment in CDH survivors supports the need for standardized long-term surveillance, particularly in children who already show pulmonary function impairment or other clinical signs consistent with respiratory limitation.

**Supplementary Information:**

The online version contains supplementary material available at 10.1007/s00383-026-06316-7.

## Introduction

Congenital diaphragmatic hernia (CDH) is a rare developmental anomaly with a prevalence of 2.3 cases per 10,000 births [1]. It is characterized by incomplete closure of the diaphragm, resulting in the herniation of abdominal organs into the thoracic cavity [2]. Although advances in clinical management have improved neonatal outcomes and overall survival, a long-term disease burden remains common among survivors [3]. 

Pulmonary pathophysiology, specifically lung hypoplasia and pulmonary hypertension, plays a decisive role in this context. Pulmonary hypoplasia is characterized by disrupted branching morphogenesis, immature lung tissue, and reduced alveolar units, resulting in impaired gas exchange. Concurrently, pathological vasculogenesis leads to decreased vascular density, vascular remodeling, and pulmonary vascular dysfunction. Furthermore, fetal cardiac hypoplasia, particularly affecting the left ventricle, predisposes to postnatal pulmonary hypertension. These alterations significantly contribute to long-term morbidity, emphasizing the need for continued surveillance and targeted interventions. Incomplete diaphragmatic formation in CDH is associated with a spectrum of cardiopulmonary developmental anomalies that significantly impact early mortality, morbidity, and quality of life [4]. 

To explain this complex constellation of developmental anomalies, the dual-hit hypothesis proposes that these cardiopulmonary malformations result from a global embryopathy driven by aberrant smooth muscle cell signaling from the pleuroperitoneal folds, rather than solely mechanical compression [2]. 

Current literature consistently reports abnormal indices of lung function among individuals with CDH [3]. Across multiple studies, spirometry results have demonstrated reductions in forced expiratory volume in one second (FEV1), forced vital capacity (FVC), and FEV1/FVC ratio in children and adolescents with CDH compared to healthy controls [5–11]. Furthermore, full-body plethysmography studies, such as those by Ijsselstijn et al. [12] and Laviola et al. [13], have revealed alterations in lung volumes, including increased residual volumes and decreased tidal volumes in CDH patients. Importantly, these abnormal lung function indices have been clinically associated with adverse health outcomes and reduced health-related quality of life (HRQoL) [14]. 

Yet, most studies investigating long-term cardiopulmonary outcomes in CDH patients encompass relatively small patient cohorts and exhibit significant heterogeneity with respect to disease severity, clinical management strategies, and patient age at enrollment [3]. Consequently, available data on the prevalence of obstructive or restrictive lung diseases as well as specific diagnoses such as asthma is limited and inconsistent. Moreover, there is paucity of data on the longitudinal course of lung function impairments and on risk factors for the observed cardiopulmonary morbidity.

The objective of this prospective long-term cohort study was to address this gap in knowledge, by defining the characteristics and predictors of lung function impairments in a large cohort of CDH patients utilizing both pulmonary function tests (PFTs) and advanced imaging techniques.

## Methods

### Compliance with ethical standards

All procedures performed in this study adhered to the ethical standards of the institutional research committee; the Ethic Commission II, Medical Faculty Mannheim, Heidelberg University, and ethical approval was obtained (ID 2022 − 626).

### Study population and data acquisition

All CDH patients who underwent PFTs between 2013 and 2023 were enrolled in the study. As spirometry relies on active patient participation, it was not performed until the age of 5 years. In addition to the spirometry, lung volumes were measured using body plethysmography. Furthermore, MRI (in patients with ECMO treatment or those capable of undergoing MRI without sedation) or CT (in all other patients) examinations were routinely performed at ages 2 and 10 years. MRI and CT scans were used to quantify thoracic morphology and deformities, as performed previously [15]. To investigate the relationship between pulmonary function and thoracic morphology, only imaging studies performed within a 6-month interval preceding or following PFT were included in this analysis. Patient demographics and treatment details were extracted from medical records. Defect size was classified according to the Congenital Diaphragmatic Hernia Study Group (CDHSG) classification system [16]. This study employed a prospective design, with data collected systematically at various time points according to the established follow-up protocol for CDH patients.

### Interpretation of PFT data

According to the current consensus recommendations from the European Respiratory Society (ERS) and American Thoracic Society (ATS), the following criteria were used to determine whether a maximal effort was achieved and acceptable measurements were obtained: an immediate start of forced expiration to ensure that the FEV1 results from a maximal effort, a sharp rise in forced expiration until peak flow was reached and a complete expiration to ensure attainment of a true FVC [17]. Measurements that did not meet one of these criteria were excluded from the analysis.

To investigate the association of pulmonary function values with disease- and therapy-related factors within the cohort, only the most recent PFT for each patient was considered, utilizing the largest prebronchodilator values for FVC, FEV1, and total lung capacity (TLC) if multiple same-day measurements were available. Lung function values were interpreted using reference data from the Global Lung Function Initiative (GLI): restrictive ventilatory impairment was defined as a TLC below the lower limit of normal (LLN) that represents the 5th percentile of values expected in a healthy reference population. An obstructive ventilatory impairment was defined by a FEV1/FVC ratio, also known as the Tiffeneau Index (TI), below the LLN [18, 19]. 

The z-scores of FEV1 and TLC were used to determine the degree of lung function impairment, forming the basis of a three-level classification system, as suggested by the ERS and the ATS: [18]

- Mild: Z-score between − 1.65 and − 2.5.
- Moderate: Z-score between − 2.5 and − 4.
- Severe: Z-score <-4.

### Statistical analysis

Statistical analysis was performed using GraphPad Prism version 10.1.0. (GraphPad Software, San Diego, CA, USA). Group comparisons were performed using the Mann-Whitney-U-test. To quantify the strength of the correlation between two quantitative variables, Spearman’s rank correlation coefficient was applied. A multiple linear regression analysis was performed to develop a predictive model for pulmonary function. P-values < 0.05 were considered statistically significant.

## Results

### Patient demographics

Over the 10-year study period from 2013 to 2023, a cumulative total of 338 PFTs was recorded in a cohort of 260 patients diagnosed with CDH at the Mannheim University Hospital. Of these, 221 (85.0%) presented with left-sided CDH. 90 (34.6%) received ECMO therapy. Thoracoscopic closure was performed in 40 (15.5%) patients, and a diaphragmatic patch was used in 204 (78.5%) patients. The CDH recurrence rate in the present cohort was 11.9%.

For patient demographics, perinatal details, defect characteristics, and treatment details see Table 1.

**Table 1:** Demographic data and disease-specific characteristics of the 260 CDH patients

All patients (*n* = 260)		
Date of birth	Range	2000–2017
Male	n (%)	150 (57.7)
C-Section		190 (76.0)
Prenatal diagnosis		206 (80.5)
Birth weight [g]	Mean (SD)	2999 (504.3)
Gestational age [weeks]		37.58 (1.69)
**Diagnostics**		
Left-sided CDH	n (%)	221 (85.0)
Right-sided CDH		39 (15.0)
Bilateral		0 (0.0)
Eventration or hernia sac		37 (14.5)
Diaphragmatic defect		219 (85.6)
Defect Type A		33 (13.0)
Defect Type B		100 (39.5)
Defect Type C		104 (41.1)
Defect Type D		16 (6.3)
Liver Herniation		153 (60.2)
Significant Comorbidities		13 (5.0)
Fetal Relative Lung Volume (FRLV) [ml]	Mean (SD)	35.15 (14.0)
Observed-to‐expected lung‐to‐head ratio (O/E LHR) [%]		41.42 (15.3)
**Therapy**		
Inhaled nitric oxide (iNO)	n (%)	146 (56.2)
Extracorporeal membrane oxygenation (ECMO)		90 (34.6)
Laparotomy repair		218 (84.5)
Thoracoscopic repair		40 (15.5)
Primary diaphragm closure		56 (21.5)
Patch diaphragm closure		204 (78.5)
Abdominal patch		34 (13.1)
Recurrence		31 (11.9)

ml : milliliter, n : number of values, SD : standard deviation

### Lung function test results

The average age of the patients at the time they underwent PFT was 9.08 (± 3.47) years. Table 2 presents PFT data and corresponding z-scores calculated by the GLI calculator, which standardizes results considering height, weight, gender, ethnicity, and age [19]. 

**Table 2:** Pulmonary function test data from 338 examinations in 260 CDH patients

All PFTs (*n* = 338)		*n*	Mean (SD)	z-score Mean (SD)
Age at time of PFT [years]		338	9.08 (3.47)	
Body height at time of PFT [cm]		338	131.97 (19.13)	
Functional residual capacity [L]	FRC	146	2.12 (1.30)	0.73 (1.81)
Expiratory reserve volume [L]	ERV	205	0.70 (0.47)	-0.63 (2.01)
Residual volume [L]	RV	200	1.24 (0.74)	1.01 (1.37)
Vital capacity [L]	VC IN	211	1.85 (0.92)	-2.83 (1.55)
Total lung capacity [L]	TLC	205	3.18 (1.54)	-0.34 (2.07)
Forced expiratory volume in 1 s [L]	FEV1	337	1.52 (3.59)	-2.26 (1.49)
Forced vital capacity [L]	FVC	338	1.81 (4.09)	-2.09 (1.55)
Tiffeneau index	TI	337	84.84 (10.78)	-0.34 (1.51)
Forced expiratory flow at 75% vital capacity [L]	FEF 75	250	0.82 (0.48)	-1.12 (1.46)
Forced expiratory flow at 25% to 75% vital capacity [L]	FEF 25/75	250	1.49 (0.78)	-1.75 (1.45)

cm: centimeter, L :liter, PFT : Pulmonary Function Testing, n : number of values, SD : standard deviation. Sample sizes for individual pulmonary function parameters vary depending on the number of measured values that met the inclusion criteria for evaluability

In total, 63 patients (24.6%) demonstrated a TI below the LLN in their most recent PFT, indicating an obstructive ventilatory defect. Based on the z-score for FEV1, the obstruction was classified as mild in 13 (20.6%), moderate in 28 (44.4%), and severe in 14 (22.2%) cases. A discrepancy was observed in 8 patients (12.7%), who presented with normal FEV1 values while their TI values fell below the calculated LLN due to a reduction in FVC [18]. 

A restrictive ventilatory defect, defined by a TLC below the LLN, was present in 35 out of 156 patients (22.4%), who underwent body plethysmography. The severity of this restriction, based on the z-sore for TLC, was mild in 18 (51.4%), moderate in 14 (40.0%), and severe in 3 (8.6%) cases [18]. 

### Correlation between pulmonary function values and disease- and therapy-related factors

**Fig. 1:**
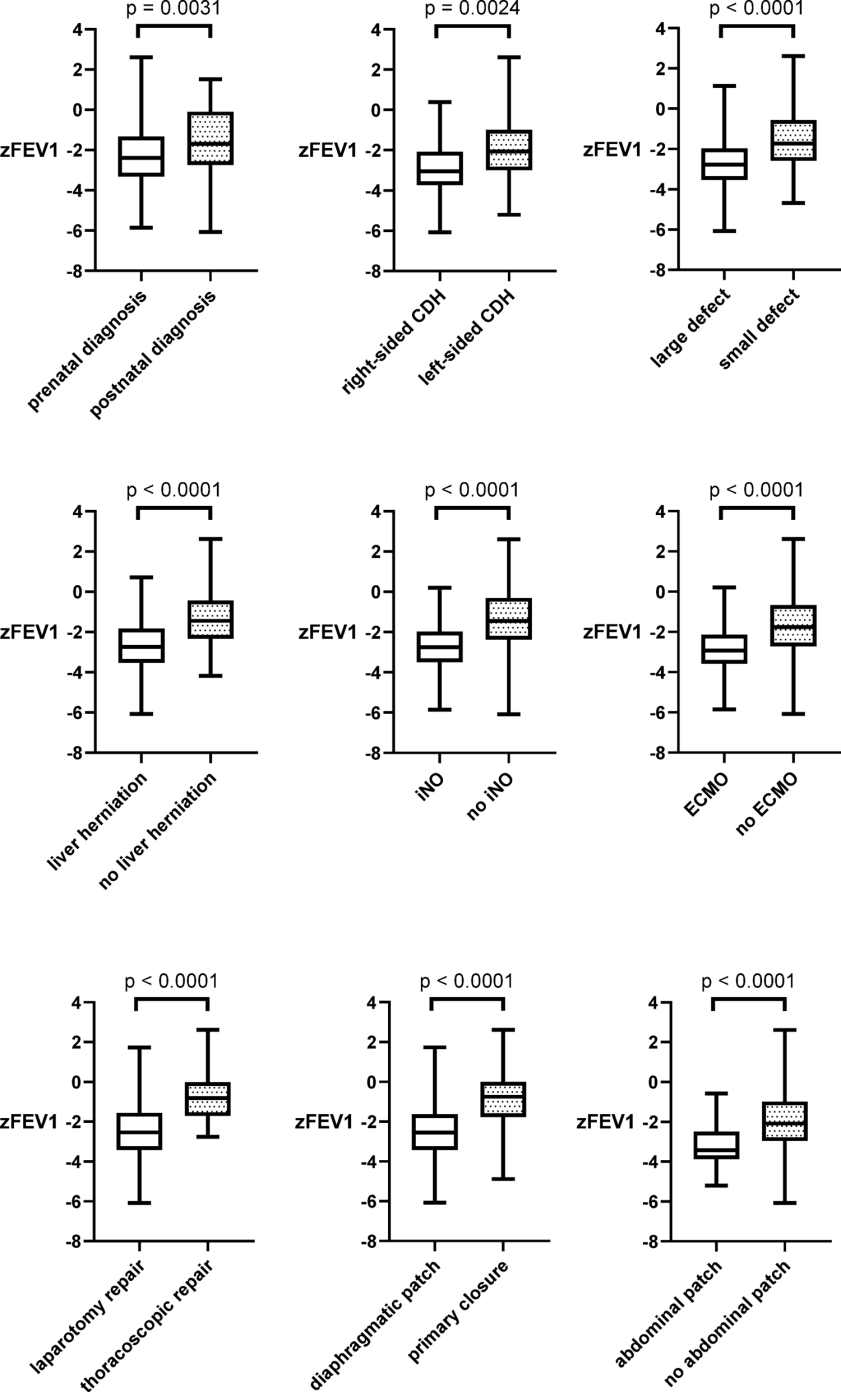
Comparison of zFEV1 scores between different patient groups (most recent PFT values of 260 patients). Defect size was classified according to the Congenital Diaphragmatic Hernia Study Group classification system [16]. For calculations, defects A and B were categorized as ‘small’, whereas defects C and D were categorized as ‘large’

To examine the relationship between pulmonary function indices and disease- and therapy-related factors, z-scores for FEV1 and TLC were compared across distinct patient cohorts. Patients with a prenatal diagnosis of CDH exhibited significantly lower FEV1 z-scores than those diagnosed postnatally (*p* = 0.0031), as did patients with right-sided CDH compared to those with left-sided CDH (*p* = 0.0024). For the present analysis, defects classified as A and B were categorized as small, whereas C and D defects were categorized as large. Patients with large defects (*p* < 0.0001) and liver herniation (*p* < 0.0001) exhibited significantly lower FEV1 z-scores. Across all therapeutic comparisons, lower FEV1 z-scores were also found for patients who had undergone NO therapy (*p* < 0.0001), ECMO therapy (*p* < 0.0001), open surgical procedures (*p* < 0.0001), diaphragmatic patch closure (*p* < 0.0001), or an abdominal patch (*p* < 0.0001). Figure 1 provides a graphical summary of all intergroup comparisons.

Z-scores for TLC differed significantly in only two comparisons: patients receiving iNO showed significantly lower values (*p* = 0.0009), as did patients with liver herniation (*p* = 0.0106).

**Fig. 2:**
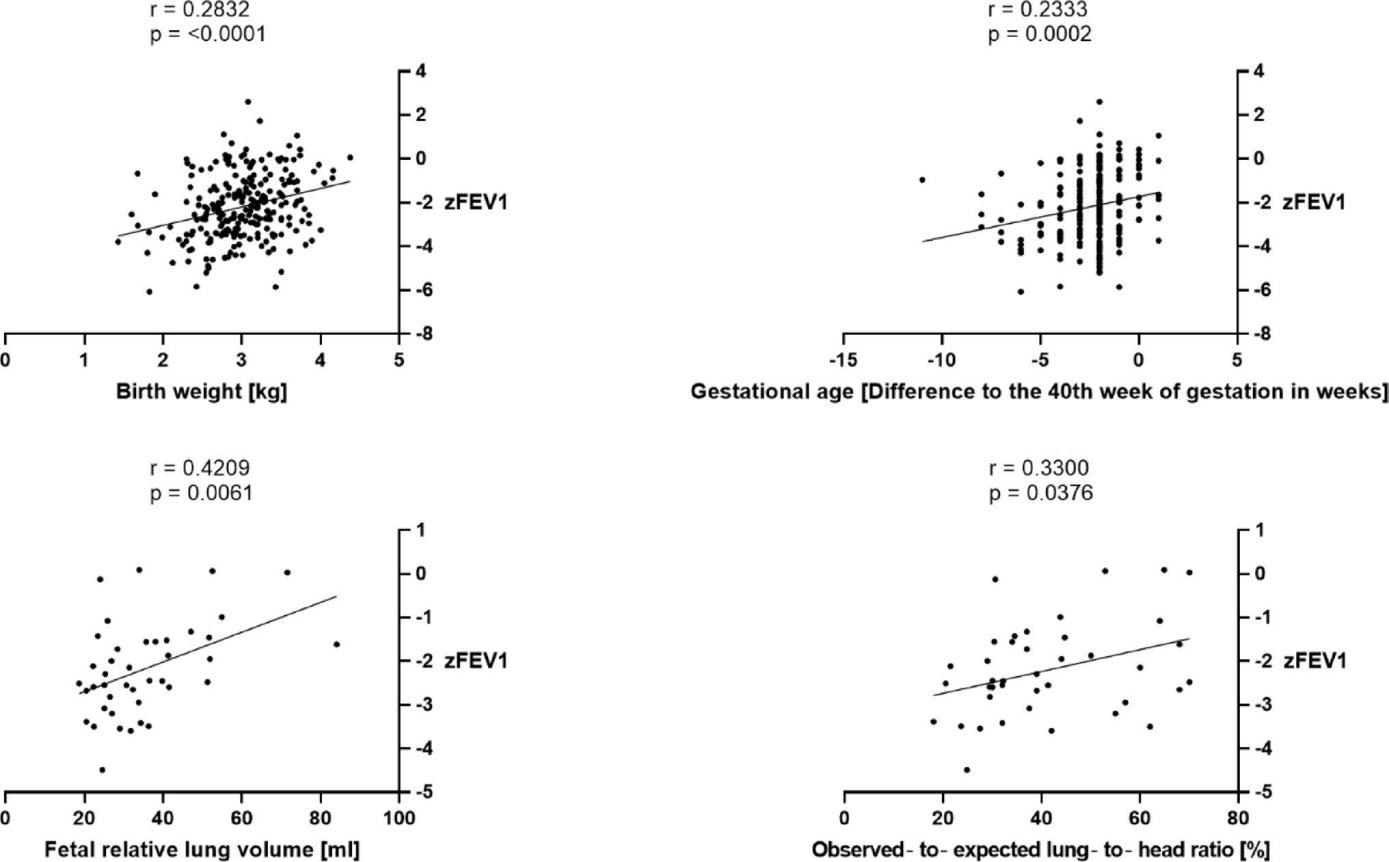
Correlation analysis between zFEV1 and perinatal variables (most recent PFT values of 260 patients)

Correlation analysis further revealed weak associations between pulmonary function and prenatal data, birth weight, and size, according to the definition by Swinscow [20]. As illustrated in Fig. 2, positive correlations were observed between z-scores for FEV1 and gestational age (*r* = 0.2333), birth weight (*r* = 0.2832), observed-to-expected lung-to-head ratio (o/E LHR) (*r* = 0.3300), and fetal relative lung volume (FRLV) (*r* = 0.4209). In contrast to zFEV1, the associations between perinatal parameters and zTLC did not reach statistical significance.

A multiple linear regression analysis in which weight, prenatal diagnosis, right-sided CDH, ECMO therapy, laparotomy, and defect size served as independent variables and zFEV1 as the dependent variable was conducted. Together these variables explained 32.05% of the variance in zFEV1 (R^2^= 0.3205). Birth weight was associated with an estimated increase in zFEV1 of 0.51 per 1000 g. Large defect size, right-sidedness of the diaphragmatic hernia, ECMO therapy, and open surgical CDH repair were significant predictors with negative effects on zFEV1 values. The corresponding regression coefficients and the significance of the effects are shown in supplementary Table S1.

### Correlation between pulmonary function values and thoracic morphology

Out of the 338 PFTs performed, 104 were accompanied by corresponding thoracic imaging studies within a six-month interval. These 104 datasets were obtained from 95 of 260 patients. The indices, as determined from the most recent imaging data of each of the 95 patients are summarized in Table 3. A total of 35 (38.04%) CDH patients exhibited an Haller-Index (HI) exceeding 2.7 (upper limit of normal) [21], and 13 (14.13%) exhibited an HI of 3.2 or more (cut off value for severe pectus excavatum (PE) deformity) [22]. In 54 (56.84%) CDH patients, the Correction Index (CI) exceeded the threshold of 0.1 for significant PE [23]. For left sided CDH, mean Asymmetry Index (AI) was 0.94 ± 0.07 and mean Area-Ratio (A-R) was 0.89 ± 0.11. 50 (52.63%) patients showed an AI below 0.95 or above 1.05 (lower respectively upper limit for healthy individuals) [24]. The measured A-R values fell outside these established normal ranges in 78 patients (82.11%).

**Table 3:** Summary of thoracic dimensions from 95 imaging studies in 95 CDH patients

Recording of pectus deformity		
HI	Mean (SD)	2.67 (0.59)
CI		0.14 (0.09)
HI > 3.2	n (%)	13 (14.13)
HI > 2.7		35 (38.04)
CI ≥ 0.28		7 (7.37)
CI ≥ 0.1		54 (56.84)
Recording of asymmetry		
AI*	Mean (SD)	0.94 (0.07)
A-R*		0.89 (0.11)
AI < 0.95 or AI > 1.05	n (%)	50 (52.63)
A-*R* < 0.95 or A-*R* > 1.05		78 (82.11)

AI : Asymmetry index, A-R : Area-Ratio, CI : Correction Index, HI : Haller Index, n:number of values, SD : standard deviation*Patients with left sided CDH only, exclusion of right sided CDH.

By correlating imaging results with pulmonary function significant associations were observed: children with a HI greater than 2.7 exhibited significantly lower FEV1 z-scores than those with an HI of 2.7 or less (*p* = 0.0262, -2.792 ± 1.644 vs. -2.143 ± 1.450). Patients with a CI of 0.1 or higher demonstrated significantly lower zFEV1 (*p* = 0.0100, -2.730 ± 1.464 vs. -1.972 ± 1.526) and zTLC (*p* = 0.0175, -0.646 ± 1.989 vs. -0.039 ± 1.486) compared to those with CI below 0.1. Furthermore, patients with an AI beyond the defined normal range (below 0.95 or above 1.05) showed significantly lower zTLC values than those with a normal AI (*p* = 0.0263, -0.775 ± 1.652 vs. 0.095 ± 1.918).

### Longitudinal course of pulmonary function limitations

**Fig. 3:**
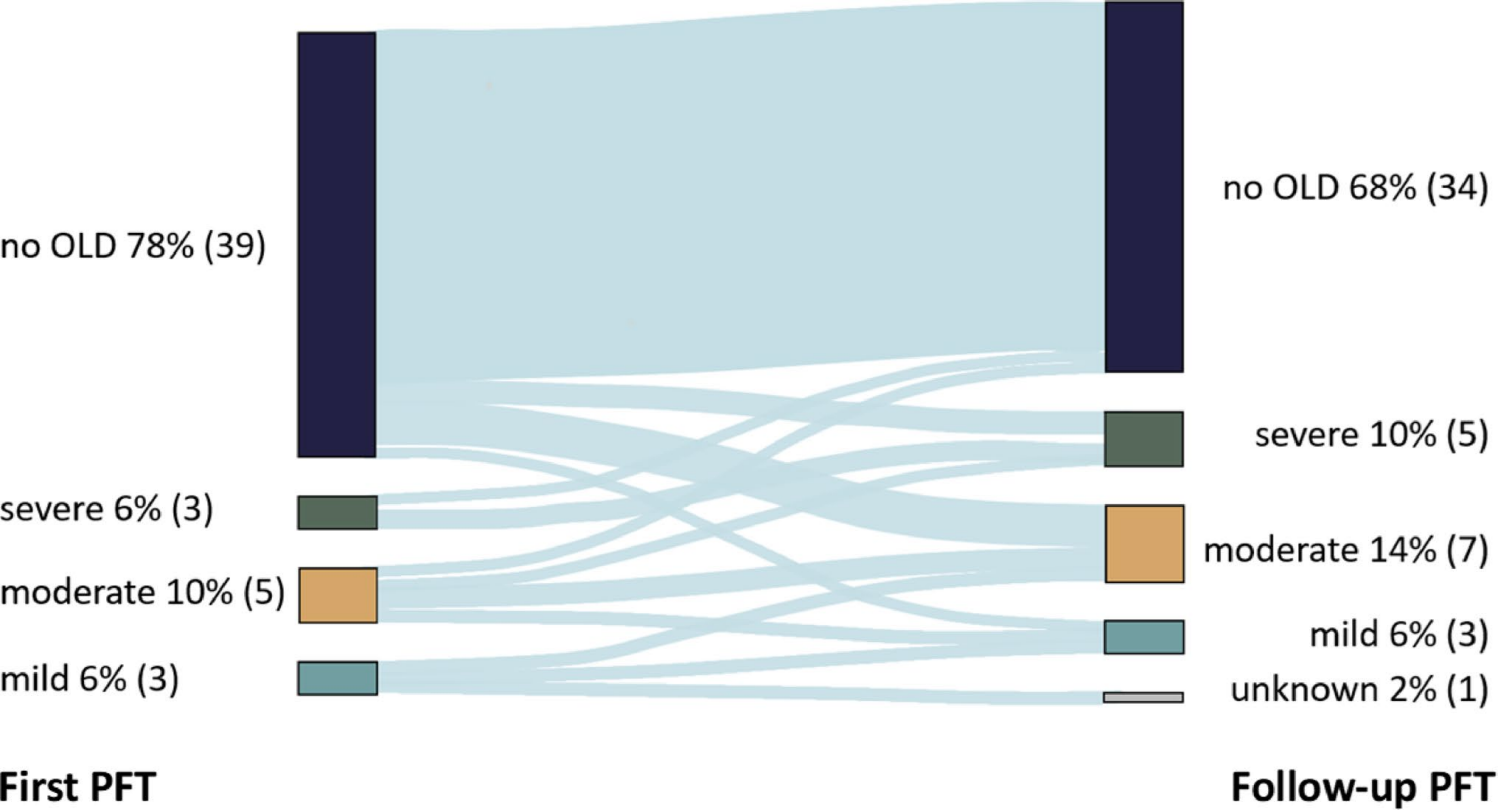
Progression of obstructive lung disease (OLD) in 50 patients with repeated pulmonary function tests (PFTs) over a 3–5-year period. No OLD = Tiffeneau Index > lower limit of normal, mild: zFEV1 between − 1.65 and − 2.5, moderate: zFEV1 between − 2.5 and − 4, severe: zFEV1 <-4. FEV1 = forced expiratory volume in one second

To assess the longitudinal trajectory of pulmonary function impairment during childhood, changes between the initial PFT and a follow-up examination were evaluated. To ensure standardized comparability, the analysis was restricted to the 50 patients who underwent follow-up PFT 3–5 years after the baseline assessment. The Sankey diagram revealed a heterogeneous disease progression over time (Fig. 3). While 41 (82.00%) patients exhibited stabilization or improvement in lung function, 9 (18.00%) developed a progressive decline accompanied by increasing severity of obstructive ventilatory impairments.

## Discussion

This study utilized comprehensive pulmonary function testing as well as and CT and MRI imaging modalities to investigate respiratory function and possible correlations in a large cohort of CDH patients, revealing significant associations among several disease severity indices, thoracic morphology, and ventilatory impairments. In brief, the study revealed that about 25% of CDH patients exhibited an obstructive ventilatory impairment and approximately 22% demonstrated a restrictive impairment, both of which correlated with disease severity. Pronounced pectus and thoracic asymmetry were linked to further reductions in lung volumes. While some patients showed improving lung function, a subgroup experienced a decline.

### CDH patients are at high risk for obstructive and restrictive ventilatory impairment

Spirometric analysis demonstrated evidence of obstructive airway disease, characterized by a decreased TI in 24.32% (*n* = 63) of the patient cohort. This finding is consistent with multiple publications documenting reduced FEV1, FVC, and FEV1/FVC ratios in similarly aged cohorts of CDH survivors [5, 6, 9, 11]. Notably, Wright et al. have also reported airflow obstruction in younger CDH patients, up to 3 years of age, utilizing infant pulmonary function testing [25]. Furthermore, studies in adolescent CDH populations (aged 13–18 years) indicate an association with mild to moderate airway obstruction and a substantial prevalence of bronchodilator responsiveness [7]. Review of the literature shows heterogenous data concerning the coexistence of CDH and restrictive lung function impairment in childhood [3, 4]. In the present cohort, however, restrictive impairment, defined as reduced TLC according to current guideline criteria, was observed in 22.44% (*n* = 25) of patients undergoing body plethysmography [18]. Moreover, 111 patients exhibited a decreased FEV1 despite a normal TI. Since this pattern can also signify restrictive impairment, it suggests that the prevalence of restrictive lung function deficits in the present cohort may be underestimated if determined solely by TLC measurements from body plethysmography [26]. The applicability of Spirometry’s to younger children further supports its potential to detect restrictive patterns beyond those identified by lung volume alone.

### CDH severity affects zFEV1

The literature identifies several key determinants of reduced lung function in CDH survivors. Prior studies have demonstrated that severity of neonatal pulmonary hypoplasia and pulmonary hypertension – defined by lower lung volumes and the use of pulmonary vasodilators – is linked to diminished PFT results [3]. Within the present cohort, comparable correlations are evident between lower o/e LHR, reduced FRLV, the application of iNO, and pulmonary function impairment. Moreover, the regression analysis conducted in the present study revealed associations between pulmonary dysfunction and several clinical variables, including prenatal diagnosis, right-sided CDH, large defect size (CDHSG Grades C and D), liver herniation, ECMO therapy, laparotomy repair, patch diaphragm closure, as well as the use of an abdominal patch. These factors are widely recognized as surrogate parameters of disease severity and are consistently reported by different authors to correlate with reduced lung function in children diagnosed with CDH [3]. 

### Potential pathophysiological mechanisms underlying pulmonary dysfunction

The observed pulmonary function impairments and their link to patient, disease, and treatment factors offer a framework for discussing possible pathophysiological mechanisms. In this context, a recent review by Zani et al. underscores the pivotal role of CDH-related structural lung abnormalities [2]. Specifically, a diminished number of alveolar units can result in a reduced overall lung capacity, thereby possibly explaining the restrictive pattern observed at high prevalence in the present cohort. Furthermore, limited airway branching could contribute to airflow limitations. combined with increased bronchoconstriction, potentially resulting from immature pulmonary epithelium and mesenchyme, as well as airway smooth muscle dysfunction, this could explain the observed obstruction.

Notably, regarding the documented association between the duration of ventilation and subsequent pulmonary function impairment, it must be emphasized that intensive care treatment itself can also contribute to pulmonary morbidity. Ventilator-induced lung injury arising from high- pressure ventilation [3] is a major concern, leading to the discontinuation of such strategies [4]. In this context, the potential benefits of ECMO use are also noteworthy, as it has been linked to improved survival in multiple series, although its long-term implications remain unclear [27, 28]. 

Although not specifically addressed in the current study, gastroesophageal reflux disease (GERD) represents another possible contributor to pulmonary dysfunction. Recurrent aspiration of gastric contents can initiate chronic airway inflammation and irritation [29]. This ongoing inflammatory process can subsequently lead to airway narrowing and remodeling. Consequently, GERD may contribute to the development of obstructive patterns.

### Progressive pulmonary function decline in CDH survivors

Regarding the longitudinal evolution of pulmonary function during growth, the current study shows that 18% of the examined CDH patients demonstrated a deterioration of pre-existing obstruction (zTI < LLN), based on FEV1. This finding aligns with the progressive decline in PFT measurements among CDH survivors reported by Lewis et al. [3] According to these authors, this trend may indicate evolving emphysema, potentially posing a risk factor for the development of chronic obstructive disease in the long term. This observation aligns with the phenomenon described by Peetsold et al., wherein alveolar size may increase following CDH repair [4]. This can lead to alveolar overdistension as the lung adapts to fill the hemithorax, potentially resulting in early airway closure, a characteristic also observed in emphysematous lungs.

The heterogeneous longitudinal trajectories observed in this cohort have direct implications for follow-up strategies. Although several markers of disease severity were associated with reduced lung function at school age, none of these factors reliably identified all patients at risk for subsequent functional deterioration. Importantly, preserved pulmonary function at a given time point did not exclude later decline, as 18% of patients developed progressive obstruction during follow-up. These findings suggest that a purely risk-stratified surveillance strategy may be insufficient. Instead, a standardized baseline pulmonary and imaging follow-up for all CDH survivors appears warranted, combined with intensified and more frequent surveillance in patients with established high-risk features or early functional impairment.

In patients without initial impairment and without major risk factors, surveillance intensity may be reduced over time; however, discontinuation of follow-up cannot be justified based on a single normal examination.

### Severity of thoracic deformity correlates with lung function impairment

With respect to the HI, prevalence of PE deformity in the current cohort is 38.04% [21] and 14.13% for severe pectus deformity [22], both exceeding rates reported in the general population [30, 31]. Applying the CI threshold of 0.1, the prevalence of substantial deformity is 56.84% [23]. aligning are with previously published long-term surveillance data from our institution [15]. 

The study revealed trends indicating lower FEV1 and TLC in patients with more pronounced pectus deformity and reduced FEV1 in those with greater thoracic asymmetry. The primary pathophysiology of PE is thought to involve a restrictive ventilatory defect [32]. Mechanisms include constraint restricting lung expansions and regional chest wall motion dysfunction, as demonstrated by oculoelectronic plethysmography.

However, a mild obstructive component may also occur due to airway compression [33]. Severe pectus deformities, particularly severe PE, can potentially compress or distort the airways, particularly during forced expiration. Finally, the altered chest wall geometry may impede respiratory muscles inefficiency contribute to both obstructive and restrictive ventilatory impairments [34]. 

### Limitations

This study has several limitations to consider. Although z-scores from a healthy reference population addressed age-related differences, a direct control group would have been preferable for comparing of the observed pulmonary function deficits with a non-affected population. Some data were extracted retrospectively and not all potential comorbidities such as asthma were considered. Additionally, PFTs rely on patient cooperation, and body plethysmography was performed only in older children. Not all patients underwent imaging within six months of pulmonary function testing. Finally, the longitudinal analysis was restricted to a sub-cohort, thus limiting the generalizability of the findings. Furthermore, this was a single-center study, which may further limit the applicability of these results to other settings. Further studies with longer follow-up periods are needed clarify the long-term pulmonary outcomes in CDH survivors.

## Conclusion

This retrospective long-term study in a large cohort of CDH patients followed up on at a large tertiary center demonstrated significant associations between disease severity, thoracic morphology, and long-term pulmonary function outcomes. The findings highlight the high prevalence of ventilatory impairments among CDH survivors. Notably, the relationship between pectus deformity severity and pulmonary function deficits underscore the importance of thoracic morphology in respiratory function.

The identified risk factors, including diaphragmatic defect size, birth weight, right-sided hernia location, the requirement for ECMO therapy, and open surgical procedures, reflects the complex interplay between congenital defects, therapy, and long-term pulmonary morbidity. The observed heterogeneity in the longitudinal course of lung function—particularly the finding that clinical stability is not guaranteed even in patients with initially preserved pulmonary function—emphasizes the need for a standardized baseline surveillance combined with individualized, risk-adapted follow-up programs for all CDH survivors.

The results suggest that CDH survivors are at increased risk for progressive pulmonary function and may benefit from early detection and management of pulmonary complications. A multimodal surveillance program, including both PFTs and imaging modalities to assess thoracic morphology, is recommended to ensure optimal long-term care. Future studies could explore the utility of the Lung Clearance Index (LCI) derived from Multiple-Breath Washout (MBW) for detecting subtle lung disease in this patient population, offering additional insights beyond traditional spirometry, as suggested by recent findings [35]. 

## Supplementary Information

Below is the link to the electronic supplementary material.

Supplementary Material 1

## Data Availability

The data that support the findings of this study are available from the corresponding author upon reasonable request.

## Abbreviations

A-R: Area-Ratio
AI: Asymmetry Index
CDH: congenital diaphragmatic hernia
CDHSG: Congenital Diaphragmatic Hernia Study Group
CI: Correction Index
CT: Computed Tomography
ECMO: Extracorporeal Membrane Oxygenation
ERS: European Respiratory Society
FEF 25/75: Forced Expiratory Flow at 25% to 75% vital capacity
FEF 75: Forced Expiratory Flow at 75% vital capacity
FEV1: Forced Expiratory Volume in one second
FRC: Functional Residual Capacity
FRLV: Fetal Relative Lung Volume
FVC: forced vital capacity
GLI: Global Lung Function Initiative
HI: Haller index
HRQoL: Health-Related Quality of Life
iNO: inhaled Nitric Oxide
LLN: Lower Limit of Normal
MRI: Magnetic Resonance Imaging
O/E LHR: Observed-to-Expected Lung-to-Head Ratio
OLD: Obstructive Lung Disease
PE: Pectus Excavatum
PFT: Pulmonary Function Test
RLD: Restrictive Lung Disease
SD: Standard Deviation
TI: Tiffeneau Index
TLC: Total Lung Capacity
