# Supplementary material for: Long-Term pulmonary function outcomes in survivors of congenital diaphragmatic hernia
Source: Pediatr Surg Int. 2026 Feb 13;42(1):87. doi: 10.1007/s00383-026-06316-7 (PMC12904957; doi:10.1007/s00383-026-06316-7)
Supplement: Supplementary file 1 — Supplementary Material 1 [file 383_2026_6316_MOESM1_ESM.docx]

**Supplementary**

Supplementary Table S1 Results of multiple linear regression analysis **for zFEV1 (**most recent PFT values of 260 patients**)**

| **Variable** | **Estimate** | **T** | **p-value** |
| --- | --- | --- | --- |
| Birth weight [kg] | 0.5109 | 3.094 | **0.0022** |
| Prenatal diagnosis | -0.3219 | 1.450 | 0.1483 |
| Right-sided CDH | -0.5675 | 2.414 | **0.0165** |
| ECMO | -0.6351 | 3.268 | **0.0012** |
| Laparotomy repair | -0.9676 | 3.992 | **<0.0001** |
| Large defect (C/D) | -0.3859 | 2.034 | **0.0431** |
